# Resources for Turkish natural language processing: A critical survey

**DOI:** 10.1007/s10579-022-09605-4

**Published:** 2022-08-26

**Authors:** Çağrı Çöltekin, A. Seza Doğruöz, Özlem Çetinoğlu

**Affiliations:** 1 University of Tübingen, Tübingen, Germany; 2 Ghent University, Ghent, Belgium; 3 University of Stuttgart, Stuttgart, Germany

**Keywords:** Turkish, Corpora, Lexical resources, NLP, Linguistics

## Abstract

This paper presents a comprehensive survey of corpora and lexical resources available for Turkish. We review a broad range of resources, focusing on the ones that are publicly available. In addition to providing information about the available linguistic resources, we present a set of recommendations, and identify gaps in the data available for conducting research and building applications in Turkish Linguistics and Natural Language Processing.

## Introduction

As in many other fields of science and engineering, the data-driven methods have been the dominant approach to natural language processing (NLP) and computational linguistics (CL) for the last few decades. The recent (re)popularization of deep learning methods increased the importance and need for the data even further. Similarly, the other subfields of theoretical and applied linguistics have also seen a shift towards more data-driven methods. As a result, availability of large and high-quality language data is essential for both linguistic research and practical NLP applications. In this paper, we present a comprehensive and critical survey of linguistic resources for Turkish.

Turkish is a language spoken by over 80 million people mainly in Turkey, also having a significant number of speakers in Cyprus, Europe, and Central Asia (Eberhard et al., 2020).^1^ It exhibits a number of interesting linguistic characteristics that are often challenging to handle in NLP applications in comparison to the well-studied languages.

As a result, the linguistic resources for Turkish are important for building practical NLP applications for a large speaker community as well as for quantitative and computational approaches to linguistics, including multilingual and cross-linguistic research. Furthermore, since Turkish is one of the largest and most well-studied languages in the Turkic language family, the resources we review below are potentially useful for language transfer in NLP applications, and as examples for resource and tool creation efforts for the other Turkic languages.

Our survey mainly focuses on currently available resources (see Aksan & Aksan, 2018, for a more historical account of Turkish corpora). We also introduce a companion webpage which we update as new linguistic resources become available.^2^ Our survey provides an overview of the available resources, giving details for the major ones, and aims to identify the areas where more effort is needed. To our knowledge, this is the first survey of its kind on Turkish resources. The most similar work is an edited volume of papers on various NLP tasks for Turkish (Oflazer & Saraçlar, 2018). Unlike our work, however, the focus is not the linguistic resources but NLP techniques and tools, and most of the contributions are updated descriptions of the research published earlier. A similar initiative to our companion website is the recently announced Turkish Data Depository (TDD) project (Safaya et al., 2022),^3^ which aims to build a repository of data and models for Turkish NLP. Our aim is collecting a more comprehensive list of pointers which can be useful for both NLP and linguistic research, while the TDD intends to store the actual data and the models for NLP with a more practical purpose.

Our focus in this survey is linguistic data, in particular, corpora and lexical resources. We do not aim to describe the research questions, methods and/or the results of these studies but focus on describing the resources in detail. We include resources that are potentially useful for NLP applications, as well as for linguistic research. We also do not focus on NLP tools explicitly, such as data-driven part-of-speech (POS) taggers or parsers and higher level tools or services that target non-technical audience such as the web-based NLP pipelines (e.g., Çöltekin, 2015b; Eryiǧit, 2014).

The main contribution of the current paper is a broad, comprehensive overview of the linguistic data available for Turkish to enable linguists and NLP researchers/practitioners to locate these resources easily. We also identify missing or incomplete resources, suggesting potential areas for future resource creation efforts. We do not only offer a static survey, but we intend to maintain a ‘living list’ of resources and a repository of publicly available linguistic data.

## Corpora

This section surveys corpora available for Turkish. We start with general-purpose, linguistically motivated corpora, followed by corpora used for more specific purposes.

### Balanced corpora

Since corpora collected from a single source (genre, domain) contain many idiosyncratic aspects of its source, the creation of balanced or representative corpora has been a major activity in computational/corpus linguistics since the earliest examples of linguistic corpora (e.g., Francis & Kučera, 1979). There are two well-known balanced corpora for Turkish, the Middle East Technical University (METU) corpus (Say et al., 2002) and Turkish National Corpus (TNC, Aksan et al., 2012).

The METU corpus is the first balanced corpus released for Turkish. The corpus consists only of written modality sampled from 14 different text types including novels, essays, research articles, travel articles, interviews, news, newspaper columns, biographies and memoirs. The corpus contains approximately 1000 documents and 1.7M tokens.^4^ The original release does not contain any linguistic annotations. However, a number of annotation projects were carried out on parts of this corpus (e.g., Oflazer et al., 2003; Zeyrek et al., 2013, both discussed in Section “Treebanks and corpora with morphosyntactic annotation”). It is available free-of-charge for research purposes after signing a license agreement.

The second balanced corpus is the Turkish National Corpus (TNC, Aksan et al., 2012). The TNC follows the design principles of the British National Corpus (BNC, Burnard, 2000). The corpus consists of 50M words from texts collected from books, periodicals, and various published and unpublished material. It also includes a small ‘spoken text’ portion that consists of political speeches and news broadcasts. The TNC contains texts from nine different domains (e.g. fiction, scientific articles, art, opinions and editorials) and includes morphological annotations. The corpus is not available for download but it is accessible through a web interface.^5^ A small part of the TNC is also used in constructing the BOUN Treebank (Türk et al., 2022, described below).

### Treebanks and corpora with morphosyntactic annotation

This section reviews primarily manually-annotated Turkish corpora with general-purpose *linguistic* annotations, as opposed to corpora annotated for a particular NLP task. The majority of the corpora discussed below are treebanks, however we also include a few other corpora with morphosyntactic annotations.

Treebanks are important resources for linguistic research and applications. Although they have been primarily used for training parsers in CL, multiple levels of linguistic annotations available in treebanks have also been beneficial for other NLP applications and linguistic research. There has been a surge of interest in creating new treebanks for Turkish in recent years. Table 1 presents the currently-available treebanks, along with basic statistics.^6^ Below, we provide a brief historical account of treebanks for Turkish.

**Table 1:** A summary of currently available Turkish treebanks

Treebank	Type	Sentences	Tokens
METU-Sabancı (Oflazer et al., 2003)	dep	5 635	56 396
ITU Web (Pamay et al., 2015)	dep	5 009	43 191
UD-GB (Çöltekin, 2015a)	dep	2 880	16 803
UD-PUD (Zeman et al., 2017)	dep	1 000	16 536
UD-BOUN (Türk Utku et al., 2022)	dep	9 761	121 214
TWT (Kayadelen et al., 2020)	dep	4 851	66 466
Turkish-Penn-CS (Yıldız et al., 2014)	con	9 560	81 419
UD-Turkish-Penn	dep	9 560	87 367
UD-Tourism	dep	19 750	92 200
UD-Kenet	dep	18 700	178 700
UD-FrameNet	dep	2 700	19 221

The numbers in the table are based on our own counts on the most recent versions of the datasets. Not all information is reported in the respective papers, and there may be mismatches between the numbers reported in the papers and the released datasets

The first Turkish treebank is the METU-Sabancı treebank (Atalay et al., 2003; Oflazer et al., 2003). The METU-Sabancı treebank is a dependency treebank including a selection of sentences from the METU corpus discussed in Section “Balanced corpora”, and includes different text types of the original resource. As an early effort with relatively low funding, the treebank had various issues with formatting and data quality (Say, 2011). Despite these issues, the METU-Sabancı treebank was the only Turkish treebank over a decade. There has been a large number of reports of fixes over the years, but most fixes remained unpublished, or even introduced other errors or unclear modifications to the annotation scheme. The most up-to-date version of this treebank is made available through Universal Dependencies (UD, De Marneffe et al., 2021; Nivre et al., 2016) repositories based on a semi-automatic conversion (Sulubacak et al., 2016) of a version from Istanbul Technical University (ITU) and hence, named UD-IMST (ITU-METU-Sabancı Treebank). Even the latest version is reported to have a large number of errors, carried over from earlier versions or introduced along the way by many automated conversion processes (see, e.g., Türk et al., 2019). Burga et al. (2017) present a conversion of the same treebank into another related framework, namely Surface-Syntactic Universal Dependencies (SUD, Gerdes et al., 2018). The paper states the intention to publish the resulting treebank, but it is not available at the time of this writing.

After a long time gap, a growing number of new dependency treebanks have recently been released. One of the new treebanks, ITU-Web treebank (Pamay et al., 2015), contains user-generated text from the web. It was annotated following the METU-Sabancı treebank annotation scheme, and later converted to the UD annotation scheme automatically. The first treebank annotated directly using the UD framework is by Çöltekin (2015a). This treebank contains linguistic examples from a grammar book to increase the coverage of different morphosyntactic constructions while minimizing the annotation effort. Two relatively larger and more recent dependency treebanks are the Boğaziçi University (BOUN) treebank (Türk et al., 2022) and the Turkish web treebank (TWT, Kayadelen et al., 2020). The BOUN treebank annotates a selection of sentences from the TNC (Aksan et al., 2012, see Section “Balanced corpora”) covering a number of different text types. The BOUN treebank is directly annotated according to the UD annotation scheme. The TWT includes sentences from the web and Wikipedia. The annotations in TwT deviate from the UD and the majority of the existing Turkish dependency treebanks.

Besides the monolingual treebanks above, there have also been a few parallel treebanking efforts. Megyesi et al. (2008, 2010) report automatically annotated parallel dependency treebanks of Turkish, Swedish and English, containing texts published in the forms of popular literature books. However, they have not been released publicly. Another early attempt of parallel treebanking is the constituency treebank described by Yıldız et al. (2014) and Kara et al. (2020b). This treebank includes translations of short sentences (less than 15 words) from Penn Treebank (Marcus et al., 1993). The UD-PUD (Zeman et al., 2017) is part of a parallel dependency treebank effort including 20 languages so far, built on sentences translated predominantly from English. The dependency annotations were performed by Google with their own annotation scheme and automatically translated to UD for the CoNLL multilingual parsing shared task (Zeman et al., 2017). A different type of multilingual treebanking effort is the UD-SAGT treebank, which annotates 2184 spoken language utterances containing Turkish–German code-switching treebank (Çetinoğlu & Çöltekin, 2019, 2022). The treebank follows the UD framework. Section “Code-switching corpora” provides further details about the underlying dataset.

Version 2.8 of the UD treebanks, released in May 2021, introduced four new Turkish treebanks from the same group. One of these treebanks is the dependency version of the Penn treebank translations (Yıldız et al., 2014). Others include a domain-specific tourism treebank, and two treebanks annotating example sentences from two lexical resources discussed in Section “Lexical Resources” below. The descriptions of the treebanks in the UD repositories indicate that all four treebanks are manually annotated. However, no formal descriptions of these treebanks have been published at the time of writing.

As described above, Turkish is relatively rich with respect to the quantity of available treebanks. However, the need for improvement in terms of the quality of annotations, establishing standards and resolving inconsistencies within and across treebanks has been emphasized by multiple researchers (see, for example Çöltekin, 2016; Say, 2011; Türk et al., 2022, for earlier discussions).

An unusual, yet potentially useful freely-available dataset with morphosyntactic annotation is ODIN (Lewis, 2006), a multilingual collection of examples from linguistics literature with interlinear glosses. Although ODIN does not include full or uniform morphosyntactic annotations, the glossed example sentences can be useful for linguistic research; they may serve as test instances with interesting or difficult linguistic constructions; and they can be converted to a treebank with less effort than that is required for annotating unanalyzed text.

There are also a few corpora that include only morphological annotations. The most popular corpus with morphological annotations is a 1M token corpus disambiguated semi-automatically. The exact procedure used for the disambiguation is unclear. The corpus was introduced by Hakkani-Tür et al. (2002), and made publicly available by later studies on morphological disambiguation (Dayanık et al., 2018; Sak et al., 2011; Yüret & Türe, 2006). Another fully manually disambiguated dataset consisting of 25098 words is reported in Kutlu and Çiçekli (2013), which can be obtained from the authors via email.

### Large-scale (unannotated) linguistic data collections

Although well-balanced, representative corpora have been at the focus of building corpora in corpus linguistics, opportunistic large collections of linguistic data have also been useful in CL/NLP tasks that require large datasets. Furthermore, the size and distribution restrictions on balanced corpora often limits their use both for NLP applications, and research on some linguistic questions (e.g., if the questions are concerned with rare linguistic phenomena). In this section, we review some of the unannotated or automatically annotated corpora that are either used in earlier literature, or publicly accessible without major limitations.

The largest Turkish corpora available are two large multilingual web-crawled datasets: supplementary data released as part of CoNLL-2017 UD parsing shared task (Ginter et al., 2017; Zeman et al., 2017), and the OSCAR corpus (Ortiz Suárez et al., 2019, 2020). Both corpora are sentence shuffled to comply with the copyright laws. The Turkish part of the CoNLL-2017 dataset contains approximately 3.5 billion words. The data is deduplicated, and automatically annotated for morphology and dependency relations. The data can be downloaded directly from the LINDAT/CLARIN repository. The OSCAR corpus is available as raw, and deduplicated versions. The Turkish section contains over 3 billion words after deduplication. The OSCAR corpus can be obtained after creating an account automatically. The publicly available data does not include any meta information, and the order of the sentences is destroyed by shuffling. However, the webpage of the OSCAR corpus includes a form to request original data without sentence shuffling.

Another popular, relatively large Turkish corpus is the BOUN corpus (Sak et al., 2008). The corpus contains approximately 500M tokens collected from two major online newspapers and other webpages. Although it is used in many studies, it is not clear how to access this corpus.

A relatively large, and easily accessible data source is the multilingual Leipzig Corpora Collection (Quasthoff et al., 2014). The Turkish section contains over 7M sentences (approximately 100M words) of news, Wikipedia and web crawl. The Leipzig corpora are also sentence shuffled. Web-crawled data also contains smaller parts crawled from Turkish-language web sites published in Cyprus and Bulgaria.

The Turkish parliamentary corpus released as part of the ParlaMint project (Erjavec et al., 2021, 2022) contains the transcripts of the Turkish parliament (2011–2021), including approximately 43M words from 303505 speeches delivered at the main proceedings of the parliament. The data also contains speaker information (name, gender, party affiliation) and automatic annotations including morphology, dependency parsing and named entities.

Another relatively large (approximately 10M words), freely accessible corpus is the Kaggle old news dataset.^7^ This is a multilingual collection from well-known news sites. The data also includes publication date of the article and the source URL of the document.

The TS Corpus (Sezer, 2017; Sezer & Sever Sezer, 2013) is also a large collection of corpora with a web interface. The collection contains some corpora released earlier (e.g., the BOUN corpus discussed in Section “Balanced corpora”) as well as sub-corpora collected by the authors. The authors report over 1.3 billion tokens in 10 sub-corpora from various text sources and various levels of (automatic) annotation. The corpus is served via a web-based query interface, and, to our knowledge, the full corpus is not publicly available for download.

Another relatively small, but potentially interesting unannotated dataset is a compilation of 6844 essays on creative writing classes by Turkish university students between 2014–2018. The essays (approximately 400K words) are published on the course webpage as PDF files.

### Corpora with discourse annotation

There are two corpora that are annotated for discourse markers in Turkish. The first one, Turkish Discourse Bank (TDB, Zeyrek et al., 2013), includes roughly 400K words across various written genres in the METU corpus (Section “Balanced corpora”). The corpus is annotated based on explicit connectives and their two arguments. The TDB is available for academic use through email. Zeyrek et al. (2018, 2010), on the other hand, focus on annotating discourse markers in the transcripts of TED talks in six languages (i.e., English, German, Polish, European Portuguese, Russian and Turkish). The Turkish corpus measures 5164 words. The annotation tasks in each language were carried out according to the Penn Discourse Treebank (PTDB) guidelines. The corpus was annotated for five discourse relation types (i.e., explicit connectives, alternative lexicalizations, implicit connectives, no relation) and five top-level senses (i.e., temporal, comparison, expansion, contingency, hypophora). The annotated corpus is freely available.

### Word sense disambiguation corpora

**Table 2:** A summary of WSD resources

Resource	Type	Additional	Samples	Sent.
METU (Orhan et al., 2007)	Lexical sample	morph, dep	26	5 385
ITU (İlgen et al., 2012)	Lexical sample	–	35	3 616
Işık (Akçakaya & Yıldız, 2018)	All-words	morph, con	7595	83 474

The ‘Additional’ column mentions additional annotations, namely, morph: POS tags and morphology, dep: dependency, con: constituency

The word sense disambiguation (WSD) task has been defined in two ways: lexical sample and all-words. The lexical sample task aims to disambiguate a restricted set of ambiguous words in their context. The all-words variant, on the other hand, disambiguates all words of a given input. Turkish has resources for both variants.

The first WSD dataset for Turkish is created as part of a SemEval 2007 task and opts for the lexical sample variant (Orhan et al., 2007). 26 unique lexical samples are tagged for their senses, and each sample is tagged in about 100 sentences. The corpus used for the annotation is the METU-Sabancı Treebank, hence the WSD dataset is already accompanied with morphosyntactic annotations. The WSD annotation adds fine-grained senses from the dictionary of Turkish Language Association (TDK), coarse-grain senses, which are a set of semantically closest fine-grained senses, and three levels of ontology. The website link provided in the paper for obtaining the resource is not accessible.

İlgen et al. (2012) also employ the lexical samples approach but choose their words among the most ambiguous words based on a frequency list (Göz, 2003). There are 35 lexical samples in total and each sample is annotated in at least 100 sentences. The corpus was collected from well-known websites on news, health, sports, and education in Turkish. The word senses come from the TDK dictionary (though the authors eliminated some senses that are infrequent in online resources). The availability of the resource is unclear.

The first all-words WSD resource for Turkish annotates a set of sentences that contains translations of Penn Treebank sentences up to 15 tokens (the treebank is described in Section “Treebanks and corpora with morphosyntactic annotation”). Akçakaya and Yıldız (2018) annotates the dependency version of the treebank as an all-words WSD resource. Therefore, the sentences also include morphosyntactic annotations. As in other resources, the sense information comes from the TDK dictionary.^8^ In total, there are 7595 unique lexical samples to disambiguate in a corpus of 83473 tokens. 77% of these unique samples are nouns, followed by verbs and adjectives. The website link provided in the paper for obtaining the resource is not accessible. The statistics for WSD resources are given in Table 2.

### Corpora of parent-child interactions

Language acquisition has been a major interest in modern linguistics, where Turkish also received a fair amount of attention because of a rather interesting learning course observed by young learners, for example, an early and error-free acquisition of case morphology (Xanthos et al., 2011). The CHILDES database (MacWhinney & Snow, 1985) contains two freely-available Turkish datasets with transcriptions of parent–caregiver interactions. The first dataset (Aksu-Koç & Slobin, 1985) contains transcripts of 54 sessions consisting of interactions with 33 children between 28 to 56 months of age. The second dataset (Altıntaş, 2005, 2012) contains transcriptions of 15 recordings with the same child between ages 16 months to 28 months. Both corpora mark speakers, and include some extra-linguistic information. The latter corpus also includes morphological annotation of a subset of the child utterances. A larger and more recent child-language dataset is reported in Moran et al. (2015). However, the Turkish section of this corpus was not released as of this writing. Rothweiler (2011) has also released a ‘Turkish-German successive bilinguals corpus’ which contains 94 longitudinal spontaneous speech samples by Turkish-German bilingual children (7–28 months-old) recorded between 2003–2008. Part of the data could be viewed for research purposes after obtaining a password.

### Social media text normalization corpora

Normalization of social media text is an important first step in many NLP applications, where ill-formed words or phrases are replaced (or associated) with their normal forms. The definition of ‘ill-formed’ text is debatable and text normalization in social media hinders analyzing social aspects of language use from a computational sociolinguistic point of view (Eisenstein, 2013; Nguyen et al., 2016). However, normalization datasets enable the use of tools created for formal/standard language, and non-destructive text normalization is also helpful in analyzing interesting aspects of non-standard language use by individuals or groups. We review corpora for normalization purposes here, for lexical resources for the same purpose, see Section “Sentiment, emotion and other application-specific lexicons”.

Eryiğit et al. (2017) report a ‘big Twitter dataset’ (BTS) for normalization which consists of 26149 tweets, as well as using IWT (see Section “Treebanks and corpora with morphosyntactic annotation”) as a source of normalization data. The BTS contains 57088 manually normalized tokens out of a total of 385568. In IWT, 5101 tokens (out of 39152 are normalized. The datasets are available from the group’s webpage after signing a license agreement. Çolakoğlu et al. (2019) introduced another normalization test set of 713 tweets (7948 tokens, 2856 normalized). The dataset is available via W-NUT 2021 Shared Task on Multilingual Text Normalization. A more recent Twitter normalization data consisting of 2000 sentences was introduced in Köksal et al. (2020). 6488 out-of-vocabulary (OoV) tokens (out of 16878) identified using lexical resources were manually annotated (below 10% of the OoV tokens are well-formed, e.g., foreign names or neologisms). The dataset is available through a GitHub repository. Besides these monolingual resources, a normalization dataset for Turkish–German is also available (Van der Goot & Çetinoğlu, 2021). This dataset is a revised version of the data from Çetinoğlu and Çöltekin (2016) for normalization by employing token-level alignment layers and adapting existing language IDs and POS tags for these new layers.

### Corpora for named entity recognition

Named entity recognition (NER) for Turkish has been studied by diverse groups of researchers with a few publicly available datasets. Tür et al. (2003) is one of the first to study NER in Turkish with a dataset compiled from newspaper articles over approximately one year (1997–1998). The dataset is annotated for ENAMEX (person, location, organization) named entity types. The dataset has been the standard benchmark for many subsequent studies, with some changes along the way. Original article reports a dataset of approximately 1M words. The version of the dataset as used by Yeniterzi (2011) consists of approximately 500K words with 37189 named entities (16291 person, 11715 location 9183 organization). This version of the data can be obtained through email. Çelikkaya et al. (2013) report three additional datasets covering different text sources, namely, a computer hardware forum, orders to a speech assistant, and Twitter. The data is also annotated for NUMEX entities (numerical expressions). Şeker and Eryiğit (2017) report an annotation effort partially based on the datasets reported in Çelikkaya et al. (2013) and Tür et al. (2003), but also annotating the IWT (described in Section “Treebanks and corpora with morphosyntactic annotation”). The datasets are available from the group’s webpage after signing a license agreement. Eken and Tantuğ (2015) also report additional 9358 tweets annotated similar to Çelikkaya et al. (2013). However, availability of this dataset is unclear.

Küçük et al. (2014) and Küçük and Can (2019) report two Twitter datasets of 2320 and 1065 tweets, respectively. These datasets are annotated for person, location, organization, date, time, money and misc (e.g., names of TV programs, music bands), and publicly available through the authors’ GitHub repositories. Another, more recent, NER data set annotating 5000 tweets was released by Çarık and Yeniterzi (2022).

### Code-switching corpora

Code-switching refers to mixing more than one language in written and spoken communication and it is quite common in multilingual settings (e.g., immigration contexts, India, Africa etc.).

Nguyen and Doğruöz (2013) and Papalexakis et al. (2014) report analyzing code-switching (e.g., Turkish-Dutch) in online fora for automatic language identification and a prediction task but this data set is not publicly available.

Çetinoğlu (2016) released a Turkish–German Twitter corpus which is annotated with language IDs. The dataset consists of 1029 tweets that are automatically collected, semi-automatically filtered, and manually annotated. Each tweet contains at least one code-switching point, the tweets are normalized and tokenized before adding language IDs. Çetinoğlu and Çöltekin (2016) added POS tag annotations to the same dataset following UD guidelines. A spoken corpus of interviews with Turkish–German bilinguals was presented by Çetinoğlu and Çöltekin (2019, 2022). The audio files are annotated with sentence and code-switching boundaries. Sentences that contain at least one code-switching point are transcribed and normalized to their orthographic representation. The resulting 2184 sentences are annotated with language IDs following (Çetinoğlu, 2017), and with lemmas, POS tags, morphological features, and dependency relations following the UD framework. The treebank version of the dataset is available in the Universal Dependencies repositories, the audio files and aligned transcriptions are available to researchers after signing a license agreement. Yirmibeşoğlu and Eryiğit (2018) worked on detecting code-switching in Turkish–English social media posts. The data is claimed to be available but it was not found on the website link suggested in the paper.

The MULTILIT project (Schroeder et al., 2015) focuses on multilingual children and adolescents of Turkish and Kurdish background living in Germany and France. The corpora they collected include Turkish oral monologues (and their transcription), and written text produced by bilingual students. A subset of the corpus is annotated with POS tags, morphological features and partial syntactic structures, as well as markers showing deviations from standard language use. The data is not publicly available. The RUEG project aims at similar goals at a larger range of age groups, and investigates bilingual speakers of Russian, Turkish and Greek background in Germany and the U.S., bilingual speakers of German in the U.S., as well as monolingual speakers of these languages in respective countries. As part of their collection there are Turkish corpora collected in Germany (1197 sentences) and in Turkey (1418 sentences), publicly available as audio files and annotated transcriptions (Wiese et al., 2020). The lemmas, POS tags, and morphological features are manually annotated, dependencies are automatically predicted. All layers follow the UD framework except the fine-grained POS tags which follow the MULTILIT project.

### Parallel corpora

**Table 3:** A selection of parallel corpora available for Turkish

Corpus	Text type	Languages	Sentences
Bianet (Ataman, 2018)	News	English, Kurdish	61 472
Bible	Religious	Multiple (102)	48 500
EU book shop	EU texts	Multiple (48)	33 398
GlobalVoices	News	Multiple (92)	8 796
JW300 (Agić & Vulić, 2019)	Religious	Multiple (380)	535 353
OpenSubtitles	Subtitles	Multiple (62)	173 215 360
QED (Abdelali et al., 2014)	Educational	Multiple (225)	753 343
SETimes (Tyers & Alperen, 2010)	News	Balkan (10)	1 776 431
TED talks	Subtitles	English	746 857
Tanzil	Religious	Multiple (42)	105 597
Tatoeba	Misc	Multiple (359)	746 857
Wikipedai (Wołk & Marasek, 2014)	Wikipedia	English, Polish	175 972
infopakki	Informational	Multiple (12)	50 909

The third column lists the languages in each corpus (numbers include Turkish), for massively parallel corpora Turkish may not be aligned to all languages. The number of sentences indicates the number of Turkish sentences in the particular corpus. The number of actual aligned sentences vary depending on the target language. All numbers are based on the corpora as available from OPUS parallel corpora collection http://opus.nlpl.eu/

Parallel, aligned corpora in multiple languages are essential for machine translation (MT) as well as multilingual or cross-lingual research. A number of parallel corpora including Turkish have been reported in some of the earlier works on MT between Turkish and mainly English (e.g., Durgar et al., 2010, 2019; Oflazer et al., 2018). Similarly, shared tasks which included Turkish as one of the languages, such as two IWLST shared tasks (Cettolo et al., 2013; Paul et al., 2010), and WMT shared tasks between 2016 and 2018 (Bojar et al., 2016), also provided data for use during the shared tasks. However, none of these resources are available, nor are there clear procedures to obtain these datasets. In this review we only list the resources available (for at least for non-commercial, research purposes) in detail.

Almost all publicly available parallel corpora that include Turkish are available from the OPUS corpora collection (Tiedemann, 2012). A selection of publicly available corpora are listed in Table 3 (except the parallel treebanks discussed in Section “Treebanks and corpora with morphosyntactic annotation”). The table does not list corpora of public software localization texts and some of the other small corpora available through OPUS. The sizes, text types and the target languages vary considerably. This list of resources, to our knowledge, are not used widely by researchers interested in machine translation to/from Turkish.

Another active area of machine translation is translation between Turkic languages (e.g., Altıntaş, 2001; Hamzaoğlu, 1993; Gilmullin, 2008; Gökırmak et al., 2019; Tantuğ et al., 2007; see Tantuğ and Adalı (2018) for a recent summary). Similar to the Turkish–English translation studies, the resources specifically built for the purpose are scarce, and even if they are reported in the literature, to our knowledge, no specific corpora build for translation between Turkic languages were released.^9^ Except for small samples in Apertium repositories (Forcada et al., 2011), the corpora build with large-scale parallel text collections (e.g., ones listed in Table 3) seem to be the only easily obtainable resource for studies requiring parallel corpora between Turkic languages.

### Corpora for sentiment and emotion

Demirtaş and Pechenizkiy (2013) introduced two Turkish datasets consisting of movie and product reviews. The movie reviews, scraped from a popular Turkish movie review site, contain 5331 positive and 5331 negative sentences. The product reviews data, scraped from an online retailer web site, consists of 700 positive and 700 negative reviews. The labels are assigned based on the scores assigned to the movie or the product by the reviewer. The datasets are available at the author’s web site.

Kaya (2013) used a balanced corpus of 400 newspapers columns from 51 journalists labeled for positive and negative sentiment. The study also reports a Twitter corpus of 123074 tweets (not labeled). Türkmenoğlu and Tantuğ (2014) also report multiple datasets, consisting of 20244 movie reviews, 4324 tweets and 101346 news headlines. The tweet dataset was annotated with three-way classes (positive, negative, neutral). Similar to other studies, the movie reviews are labeled them based on the scores assigned by the reviewers. However, it is not clear how the authors labeled the headlines corpus and used it for the presented research. Yıldırım et al. (2014) report another manually annotated Twitter dataset of 12790 tweets, labeled as positive (3541) negative (4249) and neutral (5000). None of these publications indicate the availability of the corpora introduced. Hayran and Sert (2017) present another dataset of 3200 tweets. The data is labeled (negative or positive) based on the emoticons in the messages. The dataset is available through email.

Boynukalın (2012) has investigated emotions in Turkish through two datasets. The first dataset is a translation of a multilingual emotion corpus (ISEAR, Scherer & Wallbott, 1994) into Turkish where the participants are asked to describe experiences associated with a given set of emotions (e.g., joy, sadness, anger). Although the original study describes seven emotions, the authors focused on four of them in Turkish and they have identified 4265 short texts in total. The second dataset consists of 25 fairy tales in Turkish collected across various websites on the web. The emotions in this dataset were labeled based on intensity (low, medium, high) at the sentence and paragraph levels. Demirci (2014) analyzed the emotions in a dataset of 6000 tweets, and labeled based on the hashtags they contain as anger, fear, disgust, joy, sadness, surprise. The availability of these two datasets is unclear. A more recent emotion dataset, TREMO, based on the ISEAR corpus is presented by Toçoğlu and Alpkoçak (2018). Instead of translating the original texts, Toçoğlu and Alpkoçak (2018) follow the methodology used to collect the ISEAR corpus, and collect 27350 entries from 4709 individuals describing memories and experiences related to six emotion categories. Toçoğlu et al. (2019) built a dataset consisting of 195445 tweets automatically labeled with these emotion categories based on a lexicon (see Section “Sentiment, emotion and other application-specific lexicons”) extracted from the TREMO dataset. Both of these datasets are available online for non-commercial use.

### Speech and multi-modal corpora

As in other languages, speech corpora or other forms of multi-modal datasets (e.g., video) are scarce in comparison to text corpora. The only linguistically motivated speech corpus creation effort seems to be the Spoken Turkish Corpus (STC, Ruhi et al., 2010, 2012). Although an initial sample consisting of 20 recordings, 4514 utterances and 16107 words was released in 2010, the full corpus is still not available.

Easily-accessible Turkish speech corpora are generally parts of multilingual corpus creation efforts. Notable examples include Common Voice (Ardila et al., 2020), and MediaSpeech (Kolobov et al., 2021). The Common Voice dataset is an ongoing data collection effort by Mozilla Foundation. The project collects audio recordings of a set of sentences and phrases in multiple languages. The January 2022 release includes over 68 hours of recordings from 1228 Turkish speakers. The MediaSpeech dataset includes 10 hours of speech recordings (2513 short segments less than 15 seconds each) with transcriptions from two news channels. MuST-C (Cattoni et al., 2021; Di Gangi et al., 2019) is a multilingual corpus of TED talks including Turkish transcripts, but the audio data is only in English.

The majority of the other speech datasets are collected/created within practical speech recognition/processing projects (see Arslan et al., 2020, for a recent review of Turkish speech recognition). The speech corpus introduced in Mengüşoğlu and Deroo (2001) consists of broadcast news and a set of sentences from news read by multiple speakers. Another early speech corpora collection is OrienTel-TR (Çiloğlu & Tokatlı, 2004), Turkish part of the multilingual OrienTel project (Draxler, 2003), collecting phone recordings of pronunciations of a selected set of words and phrases. Arısoy et al. (2009) report a larger dataset of broadcast news, and a dataset of 38000 hours of call center recordings is reported by Haznedaroğlu and Arslan (2014). A recent speech corpus, consisting of movies with aligned subtitles, and read speech samples are reported by Polat and Oyucu (2020). The availability of corpora listed in this paragraph is unclear.

Salor et al. (2007) report a spoken corpus of 2462 sentences, read by 193 speakers with varied ages and backgrounds. Another, similar but smaller set of recordings are available through GlobalPhone corpus (Schultz et al., 2013), which is a collection of parallel sentences from 20 languages including Turkish. Another interesting dataset where native speakers were recorded while reading parts of dialogues in the ATIS corpus (Hemphill et al., 1990) is reported in Upadhyay et al. (2018). These corpora are available for purchase through the LDC or the ELRA.

Topkaya and Erdoğan (2012) report a dataset of audio/video recordings in which 141 Turkish speakers pronounce selected numbers, names, phrases and sentences in a controlled environment. Finally, it is also worth mentioning the Turkish–German spoken code-switching treebank described in Section “Code-switching corpora” contains aligned audio recordings of Turkish–German bilinguals. Both datasets can be obtained by contacting the authors.

### Corpora for question answering

Although a highly applicable and popular area, there have been relatively few Turkish resources available for question answering (QA) until recently. Early QA work on Turkish include short lists of question–answer pairs without the context including the answer. For example Amasyalı and Diri (2005) report the use of a 524 question–answer pairs. However, to our knowledge none of these datasets are made available. Similarly, Pala Er (2009) includes 105 factoid questions and their answers as part of her thesis manuscript. Longpre et al. (2020) present a freely-available dataset containing human translations of 10000 question–answer pairs sampled from the Natural Questions dataset (Kwiatkowski et al., 2019) to 25 languages including Turkish. Another multilingual QA set released by Artetxe et al. (2020) includes 1190 human-translated question–answer pairs from Stanford Question Answering Data Set (SQuAD, Rajpurkar et al., 2016). In a more recent study, Gemirter and Goularas (2020) report both a new domain-specific dataset as well as an automatic translation of SQuAD. The availability of this dataset is unclear.

### Other corpora for specific applications

The subsections above survey the areas where a relatively large number of resources are available. In this subsection, we review other areas where there are fewer resources, either because it is a new area, or because there has not been enough interest in the Turkish CL community.

Offensive or aggressive language online has been a concern since the early days of the Internet (Lea et al., 1992). With the increasing popularity of social media, and because of the regulations introduced against certain forms of offensive language such as hate speech online, there has been a surge of interest in automatic detection of various types of offensive language. Currently, there are four Turkish corpora related to offensive language. The cyberbullying corpus by Özel et al. (2017) is a manually annotated corpus of 15658 comments collected from multiple social media sites. This dataset is not available. The corpus reported in Çöltekin (2020) is a general offensive language corpus hierarchically annotated according to OffensEval guidelines (Zampieri et al., 2019). This corpus is publicly available and consists of 36232 manually annotated tweets. In addition, two recent hate speech date sets were released by research groups at Aselsan (Toraman et al., 2022), at the Sabancı University (Beyhan et al., 2022).

Natural language inference (NLI) attracted considerable interest recently. The cross-lingual NLI dataset (XNLI, Conneau et al., 2018), includes 7500 premise–hypothesis pairs created for English, and translated to Turkish as well as 13 other languages. More recently, Budur et al. (2020) released a dataset consisting of automatic translations of Stanford NLI (SNLI, Bowman et al., 2015) and MuliNLI (Williams et al., 2018) datasets, consisting of approximately 570000 and 433000 sentence pairs, respectively. A small part of the SNLI data (250 sentence pairs) was also translated to Turkish earlier for a SemEval-2017 task (Cer et al., 2017). The data is available from the SemEval-2017 multilingual textual similarity shared task website. All NLI datasets listed above are publicly available.

Summarization datasets for Turkish are also mostly from multilingual corpora collection efforts (e.g., Ladhak et al., 2020; Scialom et al., 2020). Almost all work on summarization of Turkish texts we are aware of (e.g., Kutlu et al., 2010; Özsoy et al., 2011) rely on automatic ways to obtain texts and their summaries. However, the availability of these corpora is not clear.

Paraphrasing corpora have interesting applications such as machine translation and determining semantic similarity. Two paraphrasing corpora in Turkish are introduced in Demir et al. (2012) and Eyecioğlu and Keller (2016). The former study reports an unpublished (work-in-progress) corpus of 1270 paraphrase pairs and it can be obtained by contacting the author. The latter study reports a publicly-available corpus of 1002 paraphrase pairs which also includes human-rated semantic similarities of the sentence pairs. Another textual similarity dataset created by automatic translation of the English STS benchmark (Cer et al., 2017) is published by Beken et al. (2021).

Text categorization or topic modeling studies in Turkish often use opportunistic labeling of the topics published in newspaper sections (e.g., politics, economics, sports). Although there are many studies reporting such datasets, they are rarely made publicly available. We only note one publicly available corpus by Kılınç et al. (2017) which has become a common benchmark data for later studies. This corpus consists of 3600 news feeds (RSS) obtained from online newspapers in 6 categories.

Similar to text categorization, stylometry related studies also typically use newspaper columns scraped from online newspapers, and the corpora are not made available publicly (possibly also due to copyright restrictions). Exception we are aware of are a few datasets available from Yıldız Technical University NLP group (Amasyalı & Diri 2006; Türkoğlu et al., 2007) and the publicly available dataset of Twitter gender identification corpus by Sezerer et al. (2019), which contains 5292 users with more than 100 tweets each manually labeled for gender.

Coreference resolution is another task for which the quantity of resources available is rather small. Earlier work on coreference resolution (Küçük & Yöndem, 2007; Küçük & Yazıcı, 2008) report the use of annotated corpora without indication of availability. In the only publicly available corpus with coreference annotation, Schüller et al. (2018) annotate all sentences of METU-Sabancı treebank (described in Section Treebanks and corpora with morphosyntactic annotation) for coreference.

We also note two large multilingual COVID19-related tweet collections by Qazi et al. (2020) and Abdul-Mageed et al. (2021). The first corpus focuses on tweets geo-location in many languages. Although the number of tweets in Turkish is not specified, the total number of tweets is about half a billion. The second corpus includes 28.5M Turkish tweets with COVID-19 related keywords. Both COVID-19 datasets are available as tweet IDs. Kartal and Kutlu (2020) presents a dataset of 2287 Turkish tweets labeled whether they are worth fact checking or not. The dataset is available through a GitHub repository.

Last but not the least, we note two sign-language corpora. The first corpora of Turkish sign language was introduced by Camgöz et al. (2016), and contains sentences and phrases from finance and health domains. Eryiğit et al. (2020) present a Turkish sign language corpus with morphological and dependency annotations, as well as parallel sentences in Turkish. The availability of these two corpora is unclear. Sincan and Keleş (2020) describe a publicly available sign language corpus. However the link provided in the article is not active at the time of this writing.

## Lexical Resources

In this section we describe large lexicons and lexical networks that are built either as standalone projects or as part of multilingual collections. The majority of these lexicons also provide various levels of annotations and in multilingual cases, they usually have a mapping to the other languages of the collection.

### Lexicons, word lists

Inkelas et al. (2000) aim at creating a Turkish Electronic Living Lexicon (TELL) that reflects actual speaker knowledge. The lexicon they built consists of 30000 lexemes from dictionaries and place names. Nouns are inflected for five forms and verbs are for three, more than half also have morphological roots. All entries have phonemic transcriptions, 17500 of them also have pronunciations. Moreover, 11500 entries are annotated with their etymological source language. It is possible to search the whole lexicon via a webpage which also offers an email address to access the database. LC-STAR (Fersøe et al., 2004) is a collection of lexicons for speech translation between 13 languages including Turkish. The Turkish lexicon consists of 59213 common words (in sport, news, finance, culture, consumer information, and personal communication domains) and 43500 proper names of persons, places, and organizations. The data has been originally released via ELRA but currently it is not available in their catalog.

BabelNet (Navigli & Paolo Ponzetto, 2012) is a semantic network covering 284 languages, It is created using WordNets, Wikipedia, and machine translation. The project’s webpage offers a search interface for end users and APIs for programmers. PanLex (Kamholz et al., 2014) builds translation lexicons for over 5700 languages by utilizing their dictionaries and other multilingual resources such as WordNets. The project’s webpage lists collected lexicons and available resources for each language. However, most links for Turkish seem to be broken. While PanLex is the largest among mentioned lexicons, it should be noted that some non-Turkish entries are marked as Turkish. The lexicons, their number of lexemes, and additional annotations are summarized in Table 4.

**Table 4:** The statistics for Turkish large-scale lexicons

Lexicon	Lexemes	Additional
TELL (Inkelas et al., 2000)	30 000	phonemic transcriptions, roots, inflected forms, etymo.
LC-STAR (Fersøe et al., 2004)	104 513	phonetic transcriptions
BabelNet (Navigli & Paolo Ponzetto, 2012)	?	translations, semantic relations
Panlex (Kamholz et al., 2014)	242 635	translations

The ‘Additional’ column mentions additional annotations. ‘etymo.’ stands for etymological source

Inflectional and derivational lexicons focus on the morphosyntactic representations of words. The UniMorph project (Sylak-Glassman et al., 2015; Kirov et al., 2016) aims at building a universal schema for morphological representation of inflected forms. So far, over 120 languages are annotated (based on their webpage) with their features in a combination of automatic extractions from Wiktionary and collaborative efforts. For Turkish, there are 275460 inflected forms of 3579 unique entries (some are multiword expressions). The data is publicly available.

TrLex (Aslan et al., 2018) converts the word entries of the Turkish Language Association (TDK) dictionary into an XML format with separate fields (e.g., lemma, POS tag, origin, meaning, example) and annotates them with morphological segmentation for derivational suffixes. In addition, there is a phonological representation that encodes how entries undergo Turkish morphophonemic rules. There are 110960 entries in total. It is possible to obtain the version with morphological segmentation and POS tags through email communication with the authors.

Universal Derivations (UDer, Kyjánek et al., 2019) proposes a unified scheme for derivational morphology. The Turkish part of the project uses EtymWordNet (De Melo & Weikum, 2010) as a resource. The unified resources of 20 languages are currently available online. In the Turkish part, there are 1937 unique entries and it adds up to 7774 derived word forms. However, there are also errors (e.g., most of the derivational entries are inflectional forms).

Oflazer et al. (2004) built a multiword expression extraction tool that exploits the morphological analyzer lexicon of Oflazer (1994) for non-lexicalized and semi-lexicalized multiwords. The lexicalized multiwords collected in this study are publicly available.

Zeyrek and Başıbüyük (2019) built a lexicon of discourse connectives extracted from Turkish discourse corpora (Zeyrek et al., 2013; Zeyrek & Kurfalı, 2017; Zeyrek et al., 2018). The lexical entries are annotated with a canonical form, orthographic variants, corpus frequency and POS tags. The data is part of a publicly available multilingual connective lexicon database.

### Morphological analyzer lexicons

Since Turkish is a morphologically rich language, morphological analysis and lexical resources related to morphological analyzers have been a central component of Turkish NLP. Early attempts of building morphological analyzers date back to Köksal (1975) and Hankamer (1986). The first practical and most influential morphological analyzer is by Oflazer (1994). This analyzer has been used in a large number of studies. It is also extended by Oflazer and Inkelas (2006) to produce pronunciations as well as the written forms. However, these resources are developed using non-free Xerox tools, and their availability and license is unclear. More recently, increased availability of free finite-state tools [e.g., SFST (Schmid, 2005), HFST (Lindén et al., 2009) and Foma (Hulden, 2009)] resulted in a relatively large number of freely available morphological analyzers during the last decade. The free/open-source morphological analyzers written in conventional finite-state tools include Çöltekin (2010), Kayabaş et al. (2019), and Öztürel et al. (2019). Another popular tool is Zemberek (Akın & Akın, 2007) which is an open-source application written in Java for various NLP tasks including morphological analysis.

### WordNets and other lexical networks

A WordNet is a lexical database where lexical items (words and phrases) are grouped into synonym sets (“synsets”). All synsets are organized in a tree structure with the hypernymy relation. Some synsets also bear additional semantic relations such as antonymy. The original WordNet for English was built at Princeton University starting in 1990 (Fellbaum, 1998) and over the years WordNets have been developed for more than 200 languages (Global Wordnet Association, 2020).

The first Turkish WordNet (Bilgin et al., 2004; Çetinoğlu et al., 2018) is developed as part of the BalkaNet project (Stamou et al., 2002), which has a direct influence on the selection of synsets. As the main goal of the project was to ensure parallelism among six Balkan WordNets as well as direct mapping to Princeton WordNet and to the eight WordNets of EuroWordNet (Vossen, 1998) the majority of the synset concepts are translated from Princeton WordNet. The remaining synsets are comprised of Balkan-specific concepts and frequent Turkish words. Synonyms of translated synsets and their semantic relations are populated by exploiting the TDK dictionary. The Turkish WordNet is publicly available.

KeNet (Ehsani et al., 2018), on the contrary, follow a bottom-up approach for creating their version of the Turkish WordNet and take the concepts in the TDK dictionary as their starting point. These concepts are semi-automatically grouped into synsets and verified manually. They also exploit Turkish Wikipedia for hypernymy relations. The resulting WordNet is standalone. This is partially improved by Bakay et al. (2019) who match 4417 of most frequent English senses from Princeton WordNet to KeNet synsets. KeNet is also publicly available.

Another popular lexical network is a PropBank that annotates semantic relations between predicates and their arguments. The first example is the English PropBank (Palmer et al., 2005) and several PropBanks followed over the years, including Turkish ones. The first Turkish PropBank is annotated by Şahin and Adalı (2018) on top of the IMST dependency treebank. Later, it was adapted to the UD version of the same treebank. The annotation scheme includes numbered arguments (up to six), which correspond to the core arguments of a verb (e.g., Buyer is Arg0 for the predicate *buy*), and 14 temporary roles that represent adjunct-like arguments (e.g., DIR for direction) of a verb. The resource is available by requesting it via a license form.

Another PropBank for Turkish is constructed by Ak et al. (2018b) on top of the constituency treebank of Turkish (Yıldız et al., 2014). In this case, numbered arguments are up to four and nine temporary roles are employed. Ak et al. (2018a) compare their PropBank to that of Şahin and Adalı (2018). The same group has continued working on PropBanks and released TRopBank (Kara et al., 2020a) which employ numbered arguments up to four and a different set of semantic role labels. While the former paper has a broken link, the latter version is publicly available online. The number of sentences that are annotated and the average of arguments per predicate are provided in Table 5 for all PropBanks.

**Table 5:** Turkish PropBanks and their basic statistics.‘Avg. arg/prd’ stands for average arguments per predicate

PropBank	Sentences	Avg. arg/prd
Turkish PropBank (Şahin & Adalı, 2018)	5635	1.80
Turkish PropBank (Ak et al., 2018b)	9560	–
TRopBank (Kara et al., 2020b)	?	1.68

ConceptNet (Speer et al., 2018) is a semantic network that creates knowledge graphs from several multilingual resources such as infoboxes of Wikipedia articles, Wiktionary, and WordNets. The concepts are connected with intralingual and interlingual links. 304 languages take part in the project with varying vocabulary sizes. Turkish is in the mid-range with a vocabulary size of 65892. As a follow-up project, Speer and Lowry-Duda (2017) have developed multilingual embeddings based on ConceptNet. Both resources are available for download.

FrameNet (Baker et al., 1998) is a lexical database that structures predicates and their arguments as frames. The first FrameNet is developed for English and over the years other languages have built their FrameNets. A Turkish FrameNet was recently introduced (Marşan et al., 2021). It is designed to be compatible with KeNet (Ehsani et al., 2018; Bakay et al., 2019) and TRopBank (Kara et al., 2020b) by using the same lemma IDs. In total there are 139 frames that include 2769 synsets, which corresponds to 4080 predicates. The FrameNet is available online.

### Word embeddings and pre-trained language models

Word embeddings have gained substantial ground with the rise of neural models. As a consequence, several pretrained models for Turkish were released, as well as multilingual models. For Turkish, there are Word2vec (Şen & Erdoğan, 2014; Güngör & Yıldız, 2017),^10^ GloVe (Ferreira et al., 2016), fastText (Grave et al., 2018), ELMo (Che et al., 2018), and several BERT (Schweter, 2020) models available for download. Kuriyozov et al. (2020) created cross-lingual fastText embeddings aligned to English embeddings for five Turkic languages. The embeddings as well as the dictionaries they used for alignments are publicly available. Turkish is also part of the multilingual embeddings such as MUSE (Conneau et al., 2017), mBERT (Devlin et al., 2019), and XLM-R (Conneau et al., 2020).

### Sentiment, emotion and other application-specific lexicons

Emotion and sentiment lexicons play an important part for emotion and sentiment analysis approaches. Çakmak et al. (2012) has created an emotion words lexicon for Turkish by translating EMO20Q’s list of English emotions (Kazemzadeh et al., 2011) and adding synonyms for some translations. The total list of 197 words is not publicly available. A more recent emotion lexicon is introduced by Toçoğlu and Alpkoçak (2019), which contains scores for six emotion categories across 4966 lexical entries. The lexicon is available online for non-commercial use.

Vural (2013) has translated SentiStrength (Thelwall et al., 2012) to obtain a sentiment lexicon. SentiStrength assigns positive and negative scores to a set of words as well as creating lists of booster words, negation words, idioms, and emoticons. All lists are created also for Turkish. The paper does not provide information about the availability of the dataset.

Chen and Skiena (2014) have automatically generated sentiment lexicons for 136 languages including Turkish, using English as the source language. They used Wiktionary, Google Machine Translation API, and WordNets as mapping resources. About 60% of the words are negative in the Turkish lexicon. The dataset is accessible via the authors’ webpage.

Dehkharghani et al. (2016) utilize Turkish WordNet (Çetinoğlu et al., 2018) to create a sentiment lexicon named SentiTurkNet. They first manually label each synset with positive, negative, and neutral polarity. Then they make use of the synset mapping between Turkish and English WordNets (Fellbaum, 1998) so that by transitivity SentiTurkNet can inherit the polarity strength scores of SentiWordNet (Baccianella et al., 2010), a sentiment lexicon which is built on top of the English WordNet. The dataset is publicly available online (Table 6).

**Table 6:** The statistics for Turkish sentiment lexicons. For SentiTurkNet, each synset member is counted as one token

Sentiment Lexicon		Tokens	Polarity
Tr SentiStrength	Vural (2013)	1366	Pos (1-5), Neg (1-5)
Multilingualsentiment	Chen and Skiena (2014)	2500	Pos, Neg
SentiTurkNet	Dehkharghani et al. (2016)	21623	Pos (0-7),Neg (0-7),Neut

A normalization lexicon for social media text normalization is presented in Demir et al. (2016). The lexicon is demonstrated to provide accurate normalization, but statistics of the lexicon are not specified. The paper notes that the resource is publicly available without indicating a method for obtaining it.

## General discussion

The focus of our survey is exploring data sources for Turkish NLP applications, computational/quantitative linguistics research, as well as (digital) humanities research that may benefit from linguistic data. In this section, we list some of our observations, followed by a short list of recommendations for future efforts on creating language resources. Although we found them to be more prevalent in comparison to efforts for resource rich, well-studied languages, most of the observations and recommendations are not specific to Turkish language resource creation efforts. We believe these recommendations could particularly be useful for linguistic resource creation efforts for languages for which there are relatively few data-driven studies, and the conventions and traditions in the field are not yet well established.

### Availability and maintenance of resources

Although it is not unique to Turkish resources, we have encountered difficulties about finding and/or confirming the availability of the data sources. The locations of published resources are not always stable and/or permanent. The URLs indicating the location of the resources in papers or on the webpages of the authors or institutions are not always maintained and the resources often disappear after publication. Although our efforts to reach out to the authors/creators of the resources often yielded positive results, it is desirable to diminish these barriers to keep up with the fast-paced research community.

Another difficulty about the availability and maintenance of the resources is related to the publication traditions in other fields outside computational linguistics. In particular, most papers published in general computer science venues (e.g., in ACM conferences or journals) do not include information about the availability of their data sources. In some fields (e.g., speech processing), it is more common to make the resource available for a fee which reduces their accessibility especially for early stage researchers or researchers with limited research budgets. In addition, the majority of published resources for Turkish do not include an explicit license or ethical statement concerning collection, distribution and use of the data.

### Awareness of earlier work

Although it is not unique for the research papers in Turkish Computational Linguistics, earlier research/resources (either for Turkish or other languages) are not cited or there is only a short list of references ignoring other relevant research. This results in many repetitions and inconsistencies in the newly created resources.^11^ For example, the inconsistencies and the lack of communication during the creation of different treebanks for Turkish have been brought up by multiple researchers (see Section Treebanks and corpora with morphosyntactic annotation).

Another, related, observation is the tendency to create new resources rather than improving the existing ones. This leads to substantial effort put into the same work, without clear improvements over the earlier systems. For example, despite the fact that some of the earlier morphological analyzers reviewed in Section Morphological analyzer lexicons have been available with free licenses, a large number of new ones were created without a clear statement of difference or comparison. Similar observations can be made for other resources (e.g., WordNets) and annotation tools as well, e.g., improving existing annotation tools could be more useful than creating new tools which are often used in a single project.

Although most research in computational linguistics is publicly available, there is also a need for better communication among scholars to inform each other and collaborate on the ongoing projects, efforts and plans for building and maintaining linguistic resources. In addition, there is a need for more communication and collaboration between linguists and computational linguists for creating, annotating and analyzing language related data and resources.

### Issues about multilingual resources

There is a rapid increase in the efforts of building massively multilingual resources for various tasks and applications. We covered some of these efforts in our survey as well. By necessity, these efforts involve either opportunistic annotations (e.g., use of already existing information for other purposes, like word lists in Wiktionary), or rely heavily on crowd sourcing and/or automatic annotations. However, a potential pitfall is the lack of quality checks for these resources which do not necessarily involve linguistic expertise in each language included in the resource. For example, there are serious issues about the inflectional and derivational lexicons discussed in Section Lexicons, word lists. Although these multilingual resources are useful in many tasks, one should be aware of potential quality issues as well.

### Issues about translated resources

Like for other languages, automatic or manual translations of large datasets created originally for English are also translated to Turkish. Although this approach is interesting as it yields parallel resources, the resource created in this manner includes effects of ‘translationese’, as well as additional errors that may be introduced during the translation process. Translated datasets may even include correct translations that are not appropriate for a particular task. For example, as noted by Budur et al. (2020), the inferential relation for two English sentences may be reversed when translated to Turkish, because Turkish pronouns are gender-neutral. In general, the same type of inference in the original language may not be applicable in the translation. Similar problems are difficult to prevent with automatic translations or non-expert human translations performed without paying attention to the purpose of the dataset.

### Issues about quantity and quality

With respect to the quantity of resources, Turkish may be considered close to a ‘resource-rich’ language. For example, Turkish has the largest number of treebanks (together with English) in the Universal Dependencies repositories (as of UD version 2.10). However, most Turkish treebanks are smaller in size in comparison to treebanks in other languages, and quality and inconsistency issues have been raised in multiple earlier studies (see Section Treebanks and corpora with morphosyntactic annotation for a short discussion and pointers to relevant papers). The same trend can be observed in other types of resources as well. For example, Aksan and Aksan (2018) report partial results of a questionnaire conducted in 2011, where Turkish NLP specialists were asked to rate the quantity and quality of the available corpora on a scale of 0 to 6. The results indicate rather low judgments, 1.9 for quantity and 2.9 for quality.^12^ Although the quantity issues seem less of a problem currently, the number of linguistic resources for Turkish are still relatively low compared to well-studied European languages.

Overall, it is difficult to qualify Turkish as a ‘low-resource language’ based on the breadth and depth of the resources available. However, the resources are rather scattered across different fields, and there are issues of availability and quality. In sum, it is probably apt to classify Turkish as a ‘resource poor’ language (following the terminology used by Zaghouani (2014) for Arabic).

### Descriptions of datasets

A related problem in the publications introducing resources is the lack of sufficient descriptions. In some cases, even the basic statistics about the data are not presented or it is difficult to interpret the statistics due to unclear units of measurements. There is also a need for better descriptions of proper quality assurance procedures, metrics and inter-annotator agreements (IAA). Lack of proper linguistic glosses and translations in the provided examples also create extra barriers for readers without any Turkish background to understand and evaluate the research article and/or the data resource.

### Gaps in the existing resources

Although there are a number of sources for (social media) text normalization, we are not aware of any publications on datasets of spelling or grammar errors.^13^ Similarly, there is no known learner corpus or resources that can help second language research and practice for Turkish.

Another general area with no or little resources is semantics. Except for the lexical resources listed in Section Lexical Resources, we are not aware of any semantically annotated corpora (e.g., one that would be used for semantic parsing). There is also a lack of benchmark datasets for assessing pre-trained word or text representations (word embeddings, or pre-trained language models). So far, most linguistic resources available for Turkish aim to be domain independent. If a resource is domain-specific, it is often due to practical reasons rather than a specific interest in this particular domain. On the other hand, domain-specific data is crucial for NLP applications. Although the uses of unpublished datasets were reported in earlier literature (e.g., a corpus of radiology reports by Hadımlı & Turhan Yöndem, 2011), there is a big gap in domain-specific datasets for critical domains or sub-fields like biomedical, legal or financial NLP.

There is also a need for more systematic data collection and analysis of dialectal and sociolinguistic variation with easy-to-access language resources (Doğruöz forthcoming).

### A concise list of recommendations

The issues raised above in this section have some rather obvious solutions. Nevertheless, the concise list below may be beneficial for future resource creation efforts.

- *Publish your corpora, and publish it on permanent (or long-lasting) venues.* Beyond the value of the published data and code for reproducibility, published data allows others to study the data in ways creators of the data cannot possibly foresee. Furthermore, growing evidence suggests that the papers that publish their data get more recognition (Colavizza et al., 2020; Wieling et al., 2018). It is also important to publish the data in locations that would not disappear shortly after the publication. Our experience in this survey shows that the data shared through personal and also institutional webpages often become inaccessible as authors move to other institutions, or their research interests change. As a result, publishing the data in general repositories like Zenodo and OSF, or CLARIN repositories that are more specialized for language resources is a better choice than personal and institutional webpages. Similarly, to our experience, software development infrastructures like GitHub also provide stable locations for publishing linguistic data.
- *Describe all aspects of the corpora adequately.* As we occasionally noted above, a large number of papers we reviewed do not describe the resources introduced sufficiently. It is important for a paper to include information on aspects of the corpora such as, size, label distribution, source material, sampling method, as well as indications of annotation quality (e.g., IAA) in proper units and using proper metrics for the task at hand. Being aware of the earlier recommendations (e.g., Ide et al., 2017; Bender & Friedman, 2018; Gebru et al., 2020) for resource creation efforts and their descriptions would be useful for any annotation or curation project.
- *Be explicit about the licensing and potential ethical issues.* Although major computational linguistics venues started to require statements about legal and ethical aspects of data collection and sharing, not all the venues require such statements. It is important to be aware of the existing guidelines, such as ACM code of ethics (Gotterbarn et al., 2018), or the guidelines adapted by major CL conferences,^14^ as well as the recent discussion in the field (e.g., Rogers et al., 2021; Šuster et al., 2017). Even though the common guidelines may not fit every task, or every legal jurisdiction, being aware of potential issues, and being explicit about the legal and ethical considerations during data collection and annotation is important. The lack of clarity around these issues may also reduce the usability of the data (and hence, the recognition the creators may receive).
- *Before creating a new resource, perform a thorough literature review of the relevant research, consider improving existing resources, and collaborating with other scholars in the field.* As evidenced by the lack of citations in published papers, most resources are built from scratch, not paying attention to the lessons learned in the earlier work. The quality of linguistic resources could be improved by awareness of earlier work and more collaboration between different groups. Besides individual efforts from researchers and reviewers, a regular meeting of CL/NLP researchers and practitioners working on Turkish (and possibly Turkic languages) may help alleviate this problem. Although a number of ‘first attempts’ were made for such meetings, unlike many other CL communities, no regular/stable meeting has been established so far.
- *Contribute to multilingual resource creation efforts.* One of the issues we observed above with large-scale, multilingual resources is the lack of quality in Turkish data in these efforts. Bringing the language expertise of Turkish (computational) linguists in these projects would definitely improve the quality of these efforts, which, in turn, would be beneficial to the CL/NLP studies in Turkish.

## Conclusion

Our goal in this survey was to present a comprehensive summary of language resources NLP and computational/quantitative linguistic research for Turkish. In addition to the resources listed in our survey, we also provide a companion website (https://turkishnlp.github.io) which includes links to even more Turkish resources, and we will update it regularly. In this way, our survey and the companion website will serve as stable and sustainable resources for researchers across disciplines (e.g., linguistics, NLP) who are currently working on Turkish. In addition, researchers who are not currently working on Turkish but who need linguistic resources outside their current expertise and/or those who are interested in including Turkish in multi- or cross-lingual tasks could benefit from our contribution as well.

Besides the comprehensive overview of the resources, we have also summarized some of the common problematic issues and gaps in the field and provided a set of short suggestions for future resource creation efforts. We cautiously note that not all the problematic issues could easily be resolved by individual researchers and research groups immediately. Some of these issues require long-term collaborative efforts within the community as well as substantial support from academic funding agencies for further research. The issues we raise in this paper are based on our impression from published papers and cursory inspection of the available corpora. To understand the factors behind these issues better and propose informed solutions, future studies with in-depth analyses (e.g., through questionnaires directed to creators and users of the resources, or more systematic inspection of the available data) can be helpful. Similarly, effectiveness of the guidelines (offered in papers we cite in Section 4) may also be measured in future experimental studies.

In short, we hope that our survey and its companion webpage will serve as a useful reference for locating resources for existing fundamental and applied research and for creating future resources and projects for Turkish and/or other languages.

## Footnotes

1 Throughout this paper, we use Turkish only for referring to the language variety spoken in modern Turkey and use of this variety in other countries/regions. Hence, this count does not include other Turkic languages, including ones mutually intelligible with Turkish.

2 The web page is publicly available at https://turkishnlp.github.io. The current list was compiled mostly by our own efforts. However, we also welcome suggestions through a simple web-based form, and also through the GitHub repository associated with this URL.

3 https://tdd.ai/.

4 The numbers are based on a version obtained in 2015, which includes minor fixes to the first original release.

5 Further information and the query interface is available from https://www.tnc.org.tr/.

6 We only include manually annotated treebanks. All treebanks listed in the table are directly available for download, with the exception of ITU Web treebank, which requires a signed license agreement to be sent to the maintainers. All UD treebanks can be obtained through the project’s webpage at https://universaldependencies.org/. Automatic conversion efforts or parsed corpora are not listed in the table.

7 The corpus is not described in any earlier publication. Throughout this survey, we cite the papers describing each resource, if one is available, otherwise provide a hyperlink to the resource. Links to all available resources are provided in the companion webpage at https://turkishnlp.github.io.

8 Note that it is the same dictionary, yet different versions.

9 Except Gökırmak et al. (2019), who state the intention to release their data pending copyright clearance, most papers do not include intentions of sharing their data.

10 Also at https://github.com/akoksal/Turkish-Word2Vec without an associated publication.

11 This criticism does not refer to the creations of similar resources from multiple independent groups. As the CL and NLP become more and more data driven, we definitely benefit from more data, and well-informed and yet different approaches to the same problem.

12 The complete results of the questionnaire are not published. Hence, the wording of the questions, and the type of corpora queried are not clear.

13 A new spelling dictionary with an associated tool has been announced https://extensions.libreoffice.org/en/extensions/show/20565 during the final revisions of this paper.

14 For example, NAACL guidelines at https://2021.naacl.org/ethics/faq/ which is also adapted by some of the other major CL conferences.
